# Optimization of Chromium Removal Conditions from Tanned Leather Waste for Collagen Valorization

**DOI:** 10.3390/polym17172319

**Published:** 2025-08-27

**Authors:** Ana-Maria Nicoleta Codreanu (Manea), Daniela Simina Stefan, Lidia Kim, Ionut Cristea, Rachid Aziam

**Affiliations:** 1 Faculty of Chemical Engineering and Biotechnology, National University of Science and Technology Politehnica Bucharest, 011061 Bucharest, Romania; anamariacodreanu99@yahoo.com; 2 National Research and Development Institute for Industrial Ecology, 060652 Bucharest, Romania; lidia.kim@incdecoind.ro (L.K.); ionut.cristea@incdecoind.ro (I.C.); 3 Laboratory of Applied Chemistry and Environment, Department of Chemistry, Faculty of Sciences, Ibnou Zohr University, Agadir BP 8106, Morocco

**Keywords:** leather waste treatment, chromium removal, oxalic acid extraction, optimization, collagen hydrolysis, response surface methodology (RSM)

## Abstract

The large amounts of chrome-tanned leather waste (CLTW) produced annually can be valorized by applying circular economy principles in various fields due to the valuable substances contained (mainly collagen). The main problem for the direct valorization of these wastes is the presence in their composition of dangerous substances, such as chromium. Thus, before being used as raw material in new processes, chrome-tanned leather waste must be subjected to a preliminary stage of chromium removal. In this article, we propose to identify the optimal working conditions for the extraction of chromium ions from chrome-tanned hides in the presence of oxalic acid with various concentrations, at various temperatures and contact times, so that the degree of collagen hydrolysis is minimal. In this sense, the response surface methodology (RSM) method was used to optimize the working conditions, to maximize the efficiency of chrome extraction from the leather, and to minimize the efficiency of collagen hydrolysis: An undesirable process. To optimize both the extraction yield (%) and the degree of hydrolysis (%), the key operational variables, namely oxalic acid concentration (%), contact time (%), and temperature (°C), were systematically adjusted using the Box–Behnken design within the response surface methodology (RSM). The most favorable extraction conditions were identified at an oxalic acid concentration of approximately 7%, a contact time close to 120 min, and a temperature near 49 °C. Under these optimized parameters, the hydrolysis degree remained very low, around 0.38%, indicating minimal degradation during the process.

## 1. Introduction

The processing of animal hides is one of the oldest activities, but at the same time, it is one of the most polluting industries worldwide [1]. By processing one ton of raw leather, 200–250 Kg of finished leather product is obtained and, consequently, four times more waste (approximately 800 Kg) [2]. Even if the transformation of raw hides into finished leather leads to obtaining large amounts of waste, they can be reused in new industrial processes by applying circular economy principles [3,4]. The chemical compositions of raw hides include protein (2.5–10.5%), fat (up to 10.5%), water (up to 80%), and small amounts of mineral substances (0.35–0.5%) [5]. The proteins found in raw hide compositions are predominantly fibrous proteins (~96.5%) composed of collagen (~98%), elastin (~1%), and keratin (~1%) [6]. These useful compounds are also found in leather waste compositions, a fact that allows the reuse of this waste. For example, splits and offcuts are fat and protein sources, shavings (from trimming and leather shaping processes) are collagen sources, fleshing waste is a fat and protein source, and hair and brise are a keratin source [7].

Thus, the waste from leather processing is used in various fields. Fleshing waste has been valorized as biofuel and bioenergy [8,9], acoustic membranes [10], or active packing films [11]. Trimming waste has been used to obtain luminescent carbon dots and biochar, and hair waste has been valorized as a nanofiltration membrane or adsorbent material [12,13,14,15]. Chrome-tanned leather waste is the solid by-product formed during chromium salt tanning of hides, primarily trivalent chromium (Cr^3+^). This by-product is a major environmental concern due to the presence of heavy metals. Disposal of these wastes is a key challenge for the leather industry regarding sustainability and pollution control [16]. Chrome-tanned waste has been valorized as biooil, biochar, a precursor to anodes in lithium-ion batteries [17], or an adsorbent for wastewater treatment [18,19].

However, during the processing of raw hides into the finished product (the pretanning stage, tanning stage, and finishing stage), numerous chemical substances are used, such as anthracene, phthalates (Benzyl butyl phthalate, bis-(2-Etylhexyl) phthalate, di-butyl phthalate), formaldehyde, organotin compounds (dibutyltin oxide), azo dyes (Orange II), and chromium compounds (chromium salts) [20]. These compounds are also found in the chemical composition of waste resulting from the leather processing process. This fact prevents the direct use of leather waste in new industrial processes.

For example, chromium used in the tanning process (predominantly basic chromium sulphate) is found in chrome-tanned leather waste, CTLW, at a percentage of 2–4% [21,22]. CTLW represents the leather waste resulting from the tanning, cutting, and finishing stages and contains chromium in its composition Even if the chromium salts used in the tanning stage contain trivalent chromium, the CTLW contains both trivalent chromium (mainly Cr_2_O_3_) and hexavalent chromium provided from the finishing stages (additives, fixing agents, and pigments) [23,24]. Chromium in the hexavalent form is up to 100 times more toxic than the trivalent form [25]. Inhalation of small doses of hexavalent chromium causes allergic reactions, respiratory problems, skin ulcers, and cancer, while its ingestion leads to death [26]. Due to these aspects, it is necessary to introduce a preliminary step to remove trivalent chromium from the CTLW composition before it is used in new processes.

The proposed treatment processes were applied to chromium-bearing leather waste, with the aim of targeting trivalent chromium (Cr^3+^), the most widely used tanning species. Despite being far more toxic, hexavalent chromium (Cr^6+^) is not typically found in tanning waste except where oxidative conditions convert it. Recent studies have been of particular interest for the regulation of Cr^3+^ oxidation in tanning to prevent the production of Cr^6+^, particularly by using antioxidants and highly effective tanning agents [27].

Moreover, eco-friendly recovery and recycling of Cr^3+^ from tannery wastes have been shown to be worthy alternatives in line with circular economy principles [28].

In our previous study, the most-used methods for removing chromium from the CTLW composition were presented [29]. Trivalent chromium ions, bound to the carboxyl groups of collagens from CTLW by tanning, can be removed by acid extraction, alkaline extraction, and enzymatic extraction [24].

Alkaline extraction of trivalent chromium ions is performed with bases and inorganic oxides (CaO, MgO, NaOH, KOH, Ca(OH)_2_). The advantages of this type of extraction are the low costs and the ease of separation of the solid–liquid mixture. Extraction under these conditions has a major disadvantage due to the risk of destroying the collagen matrix in its entirety, making it impossible to valorize CTLW [30,31,32,33,34].

Enzymatic hydrolysis involves two simultaneous processes: chromium extraction with the help of enzymes (crude proteolytic enzymes, bating enzymes, and 1398 neutral protease), and the destruction of the collagen matrix at a high percentage [35,36,37].

Ion exchange in trivalent chromium ion systems is capable of effectively isolating chromium ions, but their efficiency can be reduced by the presence of competing ions and organic substances commonly found in CTLW [38]. Each method has distinct advantages and limitations, and the optimal choice must be based on a careful assessment of treatment objectives, waste composition, environmental impact, and economic feasibility [39,40].

Acid extraction of trivalent chromium ions, the subject of this study, represents one of the most-used methods of extracting chromium from the CTLW composition. This can be conducted with inorganic acids (H_2_SO_4_, HNO_3_, HCl) or with organic acids/organic acid salts (oxalic acid, acetic acid, citric acid, tartaric acid, sodium oxalate, potassium oxalate, potassium tartrate, EDTA) [41,42,43,44]. The extraction of chromium by hydrolysis in the presence of inorganic acids causes advanced degradation of collagen. By using organic chelators, chromium is extracted by substitution, protecting the collagen matrix. This method presents an advantage of reduced collagen matrix degradation from the CTLW composition during the substitution of the trivalent chromium ion with organic chelator ions. The substitution process of the trivalent chromium ion from a chromium–collagen complex was described by Malek et al., 2009 [45].

In addition to recovering chromium, this work adds value to collagen. Collagen has a hierarchical structure at multiple scales and high biocompatibility, making it particularly suitable for conversion into functional materials such as membranes and hydrogels. These forms of collagen are increasingly used in water treatment and biomedical applications. By integrating chromium extraction and collagen recovery, this process not only combats environmental pollution but also facilitates the manufacture of high-value-added biomaterials from industrial by-products [46].

This study starts from the hypothesis that the trivalent chromium ions from the CTLW composition can be effectively recovered under optimized physical–chemical processes and that this recovery can be associated with collagen valorization in order to obtain smart fertilizers with controlled release.

The aim of this study is to identify the necessary conditions to extract hexavalent chromium at maximum yield from CTLW in the presence of oxalic acid with various concentrations, at various temperatures and contact times, so that the degree of collagen hydrolysis is minimal.

To identify the optimal working conditions, the response surface methodology (RSM) method was used. Through mathematical processing of the data, the yield of chromium extraction from the leather was maximized, and the degree of collagen hydrolysis, which represents an undesirable process at this level, was minimized.

## 2. Materials and Methods

### 2.1. Experiment Design

In the experiments performed, tanning leather waste with chromium salts, CTLW, from the local leather industry in Romania was used. To remove chromium from the CTLW, oxalic acid (H_2_C_2_O_4_) ACS Reagent Grade Quality, Fluka, was used. The use of oxalic acid was indicated by the results of experiments performed in previous studies [42]. The chromium extraction efficiency and the degree of hydrolysis of the leathers were studied using an oxalic acid concentration between 2 and 8%, a contact temperature between 30 and 90 °C, and a contact time between 50 and 250 min.

### 2.2. Leather Sample Preparation and Characterization

#### 2.2.1. Preparation and Characterization

Samples of goat-tanned leather used in the chromium leaching tests were allowed to dry at room temperature until their mass remained constant. The leather samples were chopped into tiny pieces, with a diameter of less than 1 cm and a thickness of roughly 3 mm. For all the chromium extraction tests, the prepared chromium-tanned leather waste was kept at room temperature. The chemical analysis of the sample included quantitative analysis of the macronutrients (potassium, phosphorus, nitrogen), essential elements (carbon and hydrogen), and some microelements (copper, molybdenum, zinc) that are usually found in the composition of fertilizers [47]. The sample showed a content of 1298 mg/kg reported dry substance of phosphorus, 35.62 mg/kg reported at dry substance of potassium, and 11.68% reported at dry substance of nitrogen. The carbon content was 48.04% reported at dry substance, while the hydrogen content was 5.85% reported at dry substance. Additionally, small amounts of copper (10.91 mg/kg reported at dry substance), molybdenum (0.73 mg/kg reported at dry substance), and zinc (10.98 mg/kg reported at dry substance) were discovered in the sample. The analysis regarding the chromium content highlighted that a significant amount of chromium (34,950.63 mg/kg reported at dry substance) was present in the leather waste composition.

In the first and third stage, the sample was immersed in distilled water with a solid: liquid ratio (S:L ratio) of 1:11, a temperature of 35 °C, and a stirring speed of 200 rpm for 30 min. At the end of the contact time, the sample was subjected to the atmospheric pressure filtration process. In the second stage, the residue collected from the first stage was put in contact with 2, 5, and 8% oxalic acid solution, respectively, at a S:L ratio of 1:11 for 50, 150, and 250 min, respectively, at temperatures of 30, 60, and 90 °C, respectively, and at a stirring speed of 200 rpm. Afterwards, the sample was subjected to the filtration process at atmospheric pressure.

The functional group patterns of the chrome-tanned leather waste (CTLW) and dechromated CTLW samples were determined by Fourier Transform Infrared Spectroscopy (FTIR) with the aid of a Nicolet iS50 FT-IR spectrometer (Thermo Scientific, Waltham, MA, USA) equipped with a DTGS (Deuterated Triglycine Sulfate) detector.

DTGS is a thermal detector commonly used in mid-infrared spectroscopy due to its broad spectral response and stability. The spectra were recorded between 4000 and 400 cm^−1^ with a resolution of 4 cm^−1^, using the Attenuated Total Reflectance (ATR) mode.

#### 2.2.2. Calculation Procedure

To highlight the efficiency of the process, the chromium extraction yield, ɳCr extr, is presented in Equation (1):

(1) $$ȠCr\;extr=\frac{wi-wr}{wi}\times 100,$$

where ȠCr extr is the chromium extraction yield (%); ***w_i_*** is the chromium concentration in the initial leather waste sample (mg/kg reported at dry substance); and ***w_r_*** is the concentration of chromium in the residual leather waste sample obtained after the filtration process (mg/kg reported at dry substance).

The hydrolysis degree was calculated using the following equation:

(2) $$Hydrolysis\;degree(\%)=\frac{wa-wb}{wa}\times 100,$$

where ***w_a_*** represents the mass of the initial dry sample (g), and ***w_b_*** represents the mass of the dry residue after extraction process (g).

### 2.3. Box–Behnken Design Used for Experiment Development

RSM is a widely applied technique for sensitivity function analysis and determining optimal relationships among variables based on predictive models. RSM enables systematic study of the influences on a process, in a way that precise adjustments can be made to optimize the performance. To evaluate how dependent parameters reach optimal values, it is necessary to obtain equations that connect them with all independent parameters that influence the process [48,49]. For mathematical modeling of complex systems, establishing equations that relate these parameters, and optimizing working conditions, a Box–Behnken design with response surface methodology (BBD-RSM) was applied, using Design Expert 12 software. It is particularly appropriate for obtaining the maximum amount of information with high precision using the lowest number of experiments. This method enhances the efficiency of optimization processes through the methodical analysis of variable interactions and responses [50].

For the optimization of the two dependent parameters of interest, chromium extraction yield, %, and collagen hydrolysis degree, %, three levels, (−1, 0, 1), of independent parameters were established. Table 1 presents the defined levels for each variable in the Box–Behnken design (BBD), where parameter A corresponds to the oxalic acid concentration (%), parameter B represents the contact time (minutes), and parameter C indicates the temperature (°C).

### 2.4. Mathematical Modeling

The dependent parameters extraction yield (%), and hydrolysis degree (%) are modeled by a second-order polynomial equation, depending on various independent functional parameters such as: the oxalic acid concentration (%), the contact time (min), and the temperature (°C). The optimization of this process was carried out using a Box–Behnken design combined with response surface methodology, based on a set of 17 experimental runs.

Using the Box–Behnken design, the extraction yield of hexavalent chromium, %, and hydrolysis degree, %, were optimized, depending on the independent factors that influence the process (A, B and C), and a second-order polynomial model, were obtained (Equation (3)).

(3) $$Y\left(\%\right)=\beta_{0}+\Sigma \beta_{i}x_{i}+\Sigma \beta_{ij}x_{i}x_{j}+\Sigma \beta_{ii}{x_{i}}^{2}+\varepsilon$$

where *Y* (%) is the predicted response for the dependent parameters (extraction yield (%) and hydrolysis degree (%)). In the regression model, $\beta_{0}$ represents the intercept or the mean response. The coefficients $\beta_{i}$, $\beta_{ij}$, and $\beta_{ii}$ correspond to the model terms and are determined from the experimental data, as they are initially unknown. The symbols $x_{i}$ and $x_{j}$ denote the independent variables, while *ε* accounts for the residual error in the model [43].

## 3. Results and Discussion

### 3.1. Leather Sample Characterization

Chrome-tanned leather contains a high nitrogen content (up to 11%). Thus, a skin sample can be used as an alternative source of nitrogen in the process of obtaining fertilizers. The analyses regarding phosphorus and potassium contents revealed the fact that the two primary macronutrients studied are present in small quantities in the composition of CTLW (1298 mg/kg reported at dry substance of phosphorus, and 35.62 mg/kg reported at dry substance of potassium) [51].

On the other hand, the chemical analysis of the waste revealed a high content of chromium (34,950.63 mg/Kg reported at dry substance). This fact demonstrates that the direct reuse of this sample in order to obtain new products in different fields, such as agriculture [52], sensors [42], construction [53,54], and biofuels [18], is not possible without prior extraction of chromium. The maximum chromium content permitted in the composition of fertilizers is 400 mg/Kg [55]. Thus, a step to remove chromium from the composition of the studied waste is required.

The dechromation process for CTLW, with the help of oxalic acid, is based on the substitution method. This process takes place in two stages. In the first stage, the breaking of the first bond of trivalent chromium–collagen from the CTLW composition takes place. Basically, the water molecule around the first chromium atom is substituted by the ionized carboxylic group of oxalic acid. In the second stage, the breaking of the second bond of trivalent chromium–collagen takes place. The water molecule around the second chromium atom is substituted by the ionized carboxylic group of oxalic acid. Thus, a trivalent chromium–oxalic acid complex is formed, while the CTLW is dechromated [41].

**Figure 1:**
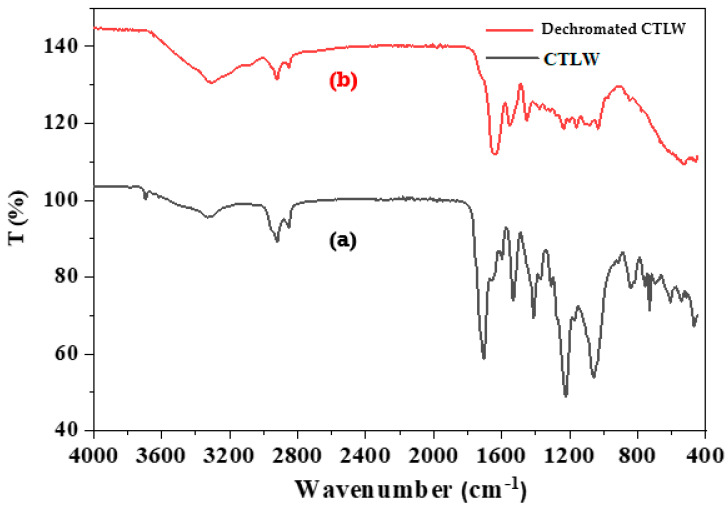
FTIR spectra of (**a**) CTLW and (**b**) dechromated CTLW.

FTIR spectra for CTLW and dechromated leather waste samples are presented in Figure 1. The figure shows the FTIR spectra of CTLW before and after chromium removal, covering a range from 4000 to 400 cm^−1^. Distinct spectral shifts confirm chromium extraction, while the specific collagen bands remain unchanged. In the high-frequency region (2900–3400 cm^−1^), both samples exhibit broad absorption bands attributed to O–H and N–H stretching, associated with amides A and B. The continued presence of these signals after oxalic acid treatment indicates that the collagen backbone remains structurally preserved.

Significant absorption bands appear between 1650 and 1500 cm^−1^ in the CTLW and dechromated CTLW samples, corresponding to amide I (C=O stretching) and amide II (N–H bending and C–N stretching). Their constant presence confirms that the collagen structure is not affected by the dechroming treatment. The presence of the absorption band near 1230 cm^−1^, attributed to amide III vibrations involving N–H bending and C–N stretching in the dechromated sample, indicates that the molecular structure of the collagen remains intact after the extraction process.

A notable difference between the two spectra appears in the low-frequency range (500–600 cm^−1^), where the CTLW sample shows a distinct band attributed to Cr-C or Cr-O stretching modes. This feature is completely absent in the spectrum of the dechromated CTLW, clearly demonstrating the elimination of chromium. The absence of this peak in the dechromated CTLW spectrum indicates the removal of chromium from the tested waste. In conclusion, FTIR spectral data confirm the successful removal of chromium from leather waste without significantly affecting the structural integrity of the collagen matrix [37,38].

### 3.2. Design of Experiments for BBD-RSM: Optimization of Extraction Yield %

#### 3.2.1. Analysis of Variance and Residuals

Optimization of the extraction yield (%) was achieved by adopting the BBD-RSM method. Polynomial models that were adopted for the response selected and the regression calculations demonstrated that there is a good fit between R^2^ predicted and adjusted for the proposed quadratic model, which proved to be statistically significant. The results obtained from the ANOVA were used to find the efficiency of the model formed.

For the identification of statistical interactions between independent and dependent variables in the response model, the statistical method analysis of variance, ANOVA, was used [56]. The variance analysis results for the extraction parameter model are presented in Table 2. According to the results shown in Table 2, the model applied shows relevant results for the three factors studied: oxalic acid concentration (%) (A), contact time (minutes) (B), and temperature (°C) (C). The data in the table indicate that the main variables (A, B, C) and their quadratic forms (A^2^, B^2^, C^2^) have a strong effect on extraction yield (%), making them the most significant elements of the experiment. Using ANOVA, interactions with significant weights and *p*-values < 0.05 were identified. From analysis of the results obtained, it was found that *p*-values above 0.05 indicate that the interactions between oxalic acid concentration and contact time (AB), as well as between oxalic acid concentration and temperature and between contact time and temperature (BC), are not significant, being of no relevance in the economics of the process [57,58]. An F-value of 86.45 confirms the statistical significance of the model, indicating a robust overall fit to the data [55]. The accuracy of the developed models is further supported by a high correlation coefficient (R^2^), which reaches 0.9911 in this case. This value suggests that the model accounts for approximately 99.11% of the variability observed in the extraction. It was found that there is a strong correlation between the coefficient R^2^ (0.9911) and the adjusted correlation coefficient $R_{aj}^{2}$ (0.9796), which confirms that the conditions necessary for the validation of the proposed model are satisfactorily met. The signal-to-noise ratio was measured using Adeq precision; a ratio with values greater than 4 is considered favorable. The values obtained for this ratio were 30.5174, which proves that the signal is considered sufficient. This model is suitable for exploring the design space (Table 2).

The quadratic equation derived for the extraction process, based on the selected input variables, is expressed as follows:

*Y* (Extraction Yield (%)) = +66.55 + 26.24 A + 10.17 B + 15.15 C + 0.5625 AB + 1.67 AC + 0.6850 BC − 4.41 A^2^ − 13.03 B^2^ − 12.08 C^2^ (4)

**Figure 2:**
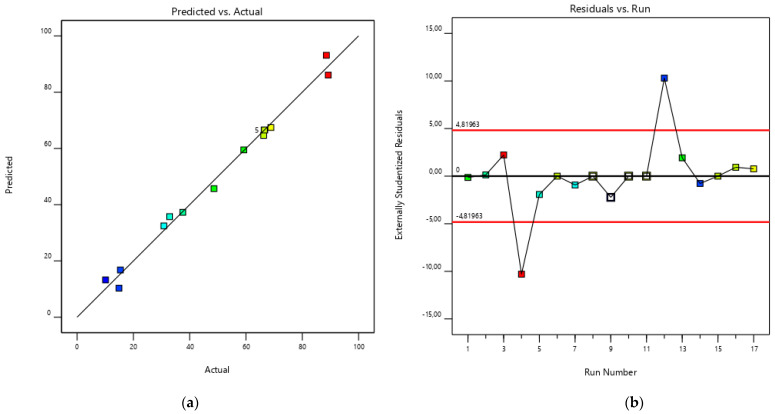
(**a**) Comparison of experimental and BBD-RSM predicted extraction yield (%) response. (**b**) Residuals study.

The model equation can be used to predict responses at specific levels of the factors. Figure 2 illustrates the standard residuals on a normal distribution. The residuals are dispersed arbitrarily near zero, showing a very close variation along the line of the first bisector. The straight line through the points demonstrates that the predicted model fits very well to the experimental data.

#### 3.2.2. Graphical Representation of Response Surfaces

**Figure 3:**
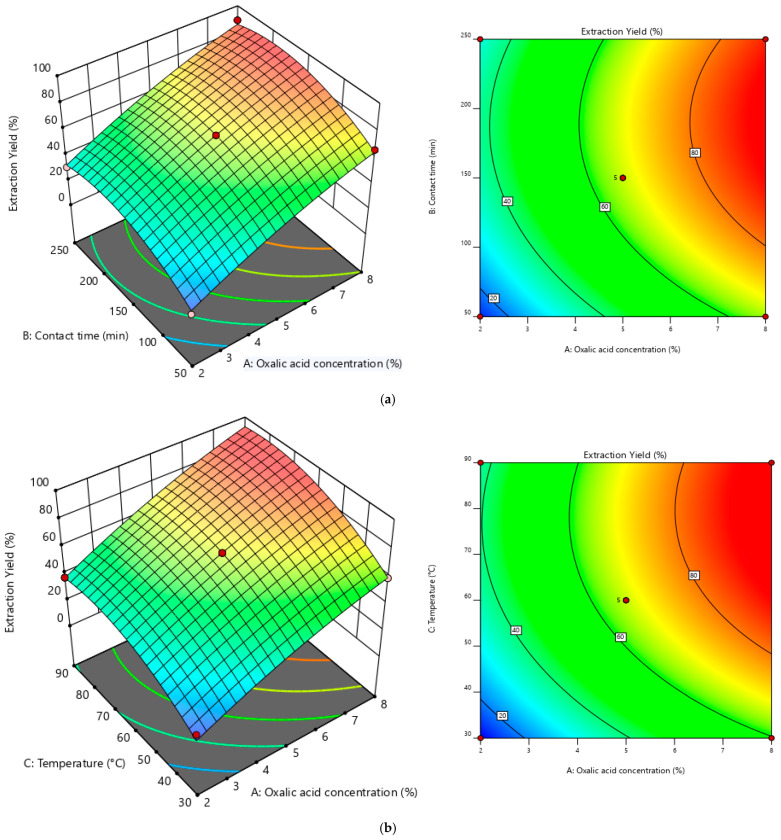
Three-dimensional response surface and contour plots of interactions. (**a**) Oxalic acid concentration versus contact time interaction. (**b**) Oxalic acid concentration versus temperature interaction.

Graphical representations in 3D coordinates of the effects of the three variables tested on the extraction yield, Y, of chromium from tanned leather (%) were generated using Design Expert 12 software and are presented in Figure 3. The plots show how response values change as a function of experimental parameters. The 3D surface plots and contour diagrams provide a clear view of the interaction between two variables at a time, while keeping the other factors at a fixed value. The three-dimensional surface graphs generated from the regression model illustrate how two experimental factors interact to influence extraction efficiency. These visual tools support the identification of optimal operating conditions by analyzing statistical patterns across different parameter combinations.

Figure 3a,b shows in 3D the simultaneous influence of oxalic acid concentration and contact time, and the influence of oxalic acid concentration and temperature, respectively, on chromium extraction yield. In Figure 3a, the concentration of oxalic acid ranges from 2 to 8%, and the contact time ranges from 50 to 250 min. In Figure 3b, the temperature ranges from 30 to 90 °C, and the oxalic acid concentration range remains constant.

To achieve an optimal extraction yield, *Y* (%), specific conditions must be met. The concentration of oxalic acid (A) should approximately range between 6.43% and 8%, while the contact time (B) needs to be maintained between 113.21 min and 250 min. Additionally, temperature (C) plays a crucial role and should be roughly set between 45.58 °C and 90 °C to ensure efficiency.

#### 3.2.3. Optimization Using Desirability Function

**Figure 4:**
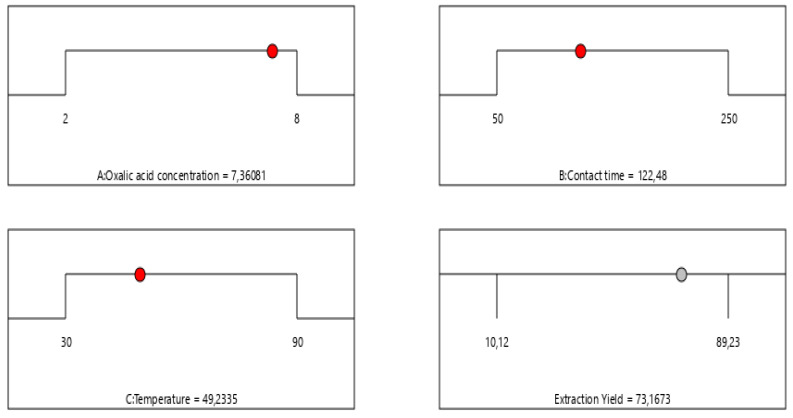
Ramps of optimal solutions with desirability equal to 1.

To determine the most favorable conditions that ensure both a high extraction yield and overall process suitability, the desirability function was applied. An overview of the criteria constraints and the optimal solutions predicted by the model is presented in Table 3 and Figure 4, highlighting the parameter settings that enhance extraction efficiency.

The developed mathematical models were used to refine the working conditions to maximize the chrome extraction yield from the tanned leather. Our findings indicate that achieving an extraction yield of approximately 73.1673% requires adherence to the following parameters: An oxalic acid concentration of 7.36061%, a contact time of 122.48 min, and a temperature close to 49.2335 °C. These parameters have been recognized as the most effective conditions to enhance extraction yield, based on model predictions.

#### 3.2.4. Data Validation

To assess the predictive accuracy of the response surface methodology, the model’s estimated chromium extraction yields were evaluated against actual experimental results across 17 trials. The comparison revealed a mean absolute error (MAE) of 1.94% and a root mean square error (RMSE) of 2.45%, demonstrating a strong correlation between predicted and observed values and confirming the reliability of the model. Model reliability was further supported by a predicted coefficient (R^2^) of 0.85, indicating a strong fit between predicted and experimental data. Residual plots confirmed the absence of systematic trends, suggesting that assumptions of homoscedasticity and model adequacy were met. Under optimized conditions such as 49.2353 °C, 122.48 min, and approximately 7.36061% oxalic acid, the experimental chromium extraction yield reached 73.1673%. It is important to note that these optimal conditions were obtained by adjusting three experimental parameters. For clarity and scientific accuracy, the values were reasonably approximated to 7% of oxalic acid, 120 min, and 49 °C. This adjustment ensures that the reported parameters remain consistent with the observed experimental evolutions, while providing a more robust and interpretable framework for practical application. These findings validate the robustness of the model and underscore its suitability for guiding process optimization.

### 3.3. Design of Experiments for BBD-RSM: Optimization of Hydrolysis Degree (%)

#### 3.3.1. Analysis of Variance and Residuals

For the optimization of the hydrolysis degree of collagen, the BBD-RSM method was also used. Polynomial models that demonstrated a good correlation between predicted R^2^ and adjusted R^2^ for the proposed quadratic model were identified. The use of the ANOVA statistical model allowed the identification of independent parameters that are important in the economics of the hydrolysis process [56]. The variance analysis results for the hydrolysis degree parameter model are presented in Table 4. Based on the results presented in Table 4, the statistical model applied to the three input variables, oxalic acid concentration, contact time, and temperature, is considered significant. The analysis indicates that temperature (C) and its quadratic term (C^2^) have the strongest influence on the hydrolysis degree (%). In contrast, other factors, such as oxalic acid concentration (A), contact time (B), their respective quadratic terms (A^2^, B^2^), and the interaction terms (AB, AC, BC), are not significant factors because their *p*-values are above 0.005.

As in the analysis of chromium extraction yield and in the case of the analysis of parameters that have an important influence on the hydrolysis process, the ANOVA model was used, the significant influence being highlighted by *p*-values < 0.05 [57,58]. The model’s F-value of 37.01 indicates the model’s significance [58]. The reliability of the developed models is demonstrated by the coefficient of determination (R^2^). In this study, an R^2^ value of 0.9794 confirms the strong agreement between the predicted and observed data. This indicates that around 97.94% of the variation in the hydrolysis degree is explained by the proposed model. Based on the results obtained, it was found that the proposed mathematical model is validated by the fact that there is a strong correlation between the coefficient R^2^ (0. 9794) and the adjusted correlation coefficient $R_{aj}^{2}$ (0.9530). The signal–noise ratio, also known as Adeq precision, has a value of 17.1528, well above 4, which demonstrates that the signal is considered sufficient. This model is suitable for exploring the design space; see Table 4.

The quadratic model equation describing the hydrolysis degree in relation to the input variables is outlined below:

*Y* (Hydrolysis degree (%)) = +94.96205 + 0.371806 A − 0.228854 B − 3.93951 C + 0.000158 AB + 0.072528 AC + 0.001841 BC − 0.306806 A^2^ + 0.000504 B^2^ + 0.036910 C^2^ (5)

**Figure 5:**
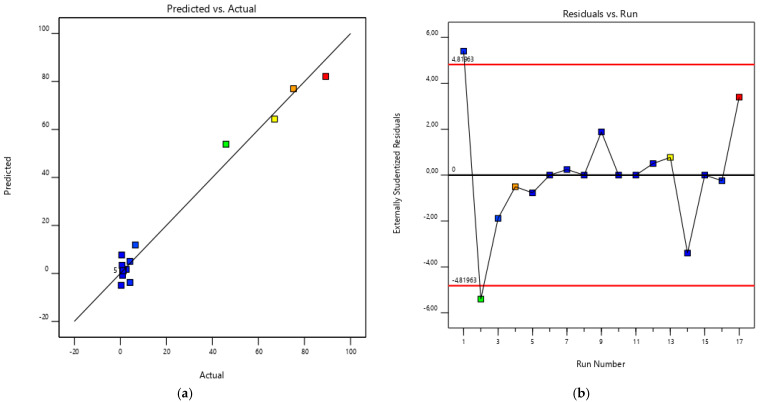
(**a**) Comparison of experimental and BBD-RSM predicted hydrolysis degree (%) response. (**b**) Residuals study.

The model equation can be used to predict responses at specific levels of the factors. Figure 5 illustrates the standard residuals on a normal distribution. Residuals are dispersed arbitrarily near zero and adopt a normal trend along the line. The straight line through the points captures the majority of the data, illustrating an approximate normal distribution.

#### 3.3.2. Graphical Representation of Response Surfaces

**Figure 6:**
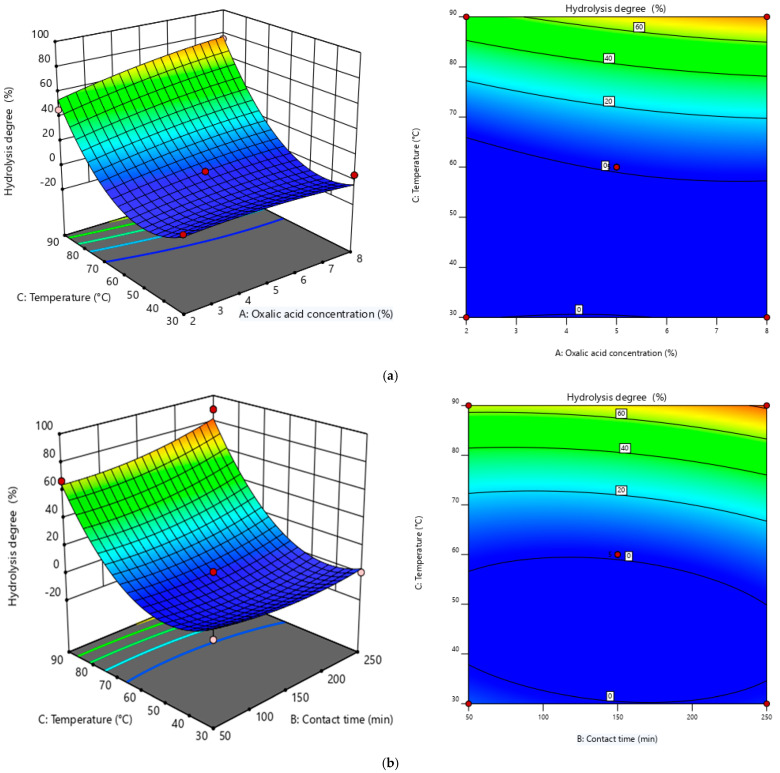
Three-dimensional response surface and contour plots of interactions. (**a**) Oxalic acid concentration versus temperature interaction. (**b**) Contact time versus temperature interaction.

Figure 6 displays contour plots produced using Design Expert 12 software, which were employed to explore how the interactions among the three studied variables influence the hydrolysis degree (*Y* %). The graphical results provide a visual interpretation of how response values are influenced by changes in the experimental parameters. The three-dimensional surface plots and contour diagrams illustrate the combined effects of two variables at a time, with the remaining factors held constant. These visual tools, generated from the regression model, help reveal the interactions between variables and support the identification of optimal hydrolysis conditions through statistical analysis across multiple parameter combinations. The influence of oxalic acid concentration versus temperature and contact time versus temperature on the hydrolysis degree is shown in 3D in Figure 5a,b, respectively. In Figure 6a, the concentration of oxalic acid ranges from 2 to 8%, and the temperature ranges from 30 to 90 °C. In Figure 6b, the contact time ranges from 50 to 250 min, and the temperature range remains constant.

To achieve an optimal hydrolysis degree, *Y* (%), specific conditions must be met. The concentration of oxalic acid (A) should approximately range between 5.4694% and 8%, while the contact time (B) needs to be roughly maintained between 144.994 min and 250 min. Additionally, a temperature increase is necessary to ensure efficiency.

#### 3.3.3. Optimization Using Desirability Function

**Figure 7:**
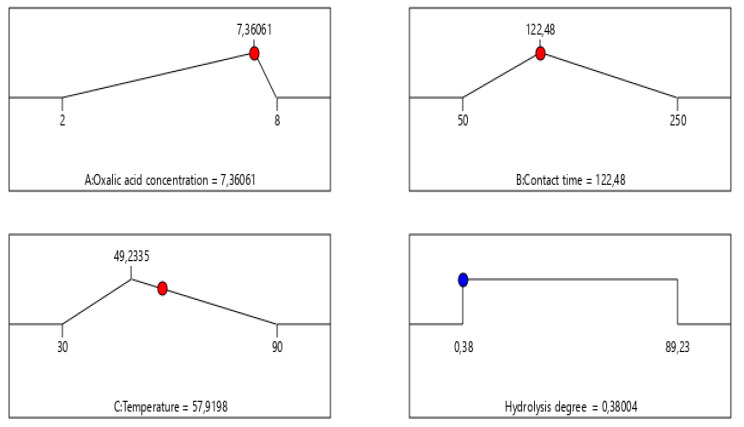
Ramps of optimal solutions with desirability = 1.

To determine the optimal operating conditions that ensure a high hydrolysis degree (%) and overall process suitability, the desirability function was used. Table 5 and Figure 7 summarize the applied constraints and highlight the optimal parameter combinations identified through this approach.

The developed mathematical models allowed us to identify the minimum degree of hydrolysis of collagen from leather waste under the working conditions for which the chromium removal efficiency is highest. Our findings indicate that achieving a hydrolysis degree of approximately 0.38004% requires adherence to the following parameters: An oxalic acid concentration of 7.36061%, a contact time of 122.48 min, and a temperature close to 57.9198 °C. These parameters have been recognized as the most effective conditions, based on model predictions, to enhance the hydrolysis degree (%).

Figure S1 visually depicts all responses, including desirability plots. A high desirability value, approaching 1, suggests that the objectives were relatively straightforward to attain, and there may be potential for even better outcomes [59,60]. The results of the response surface optimization using RSM-BBD closely matched the experimental values, validating the accuracy of the optimization process.

### 3.4. Mechanism and Comparative Study for Removing Chrome from Tanned Leather Waste

The selective extraction of trivalent chromium (Cr^3+^) from chrome-tanned leather residues is driven by a ligand exchange mechanism, wherein oxalate ions progressively displace chromium ions originally bound to collagen fibers. Oxalic acid, with its two carboxylic functional groups, exhibits strong chelating properties, enabling the formation of highly stable complexes such as Cr(C_2_O_4_)_3_^3−^. This facilitates the migration of chromium into the aqueous phase while preserving the structural integrity of the leather matrix.

In the present study, the optimal conditions were identified as a temperature of 49 °C and an oxalic acid concentration of 7% for 120 min, resulting in a chromium recovery rate of 73% and minimal collagen degradation (0.38%). These results align with prior findings by Świerczek et al. (2025), who reported an extraction efficiency of 63.1% under milder conditions (43.6 °C and 1.34% oxalic acid), with collagen hydrolysis limited to 0.70% [39]. This method offers a sustainable alternative to more aggressive extraction techniques by significantly minimizing the risk of generating hexavalent chromium (Cr^6+^), a highly toxic species that is harmful to both human health and the environment. Furthermore, the use of oxalic acid aligns with circular economy principles by promoting the recovery of chromium and the potential reuse of collagen from leather industry waste. Although oxalic acid may exhibit lower extraction efficiency compared to synthetic chelating agents like EDTA, it stands out for its superior biodegradability and reduced environmental impact [39,61].

The extraction approach proposed in this study was assessed in comparison with other research efforts that employed organic acids for chromium removal. However, many of these studies did not explicitly address the dual objective of recovering chromium while maintaining the integrity of the collagen matrix.

Numerous chromium extraction techniques have been documented, each characterized by distinct process conditions and varying impacts on collagen integrity. One approach involving citric and oxalic acids at 50 °C over an 8 h period achieved a chromium extraction rate of 78%; however, collagen underwent significant degradation when temperatures exceeded 60 °C [39]. In a separate investigation, a combination of sulfuric and oxalic acids in equal proportions was applied at 40 °C for 12 h, resulting in 96% chromium removal while simultaneously enabling the isolation of collagen with high purity [62].

The optimization performed using BBD-RSM revealed that the hydrolysis model produced a lower predicted R^2^ value (0.67) compared to the chromium extraction model (0.85). This discrepancy is consistent with findings in the literature and reflects the inherent complexity of protein degradation mechanisms. Collagen hydrolysis involves heterogeneous reactions influenced by the structural variability of the protein matrix, making it more challenging to model accurately. Hangri et al. (2025) also mentioned similar findings and emphasized the limited predictive power of enzymatic hydrolysis models in light of the complex, nonlinear nature of interactions among enzyme concentration, substrate supply, and reaction time [63].

To prove that the mathematical model describes reality well, the kinetics of the dechroming process were experimentally carried out under the optimal conditions obtained from the mathematical model: An oxalic acid concentration of 7%, a contact time of 120 min, and a temperature close to 49 °C. Under these working conditions, a chromium extraction yield of 97.6% and a degree of collagen hydrolysis of 0.4% were obtained. This proves that the mathematical model describes the process very well.

## 4. Conclusions

Optimization of the chromium extraction process and collagen hydrolysis was achieved using a Box–Behnken design with response surface methodology (BBD-RSM). Based on the mathematical models, two regression equations were identified that describe the relationship between independent parameters (initial concentration, contact time, and temperature) and dependent parameters (chromium extraction yield and degree of collagen hydrolysis). This research effectively applied response surface methodology (RSM) to optimize the extraction of chromium from leather waste using oxalic acid as an environmentally friendly leaching agent. The model exhibited strong predictive performance, as reflected by minimal deviation between predicted and experimental values (MAE = 1.94%, RMSE = 2.45%) and a robust coefficient of determination (R^2^ = 0.85). These validation metrics confirm the model’s reliability and its suitability for simulating and optimizing the extraction process.

It was observed that the optimum chromium extraction yield is obtained in the presence of oxalic acid, which proved effective in preliminary studies. This maximum yield is obtained with an initial concentration of 7%, a contact time of 120 min, and a temperature of around 49 °C. Under these conditions, the degree of hydrolysis remains minimal at around 0.38%.

The reliability of the proposed models is demonstrated by their correlation coefficient (R^2^), which reaches 0.9911 for chromium extraction yield and 0.9794 for degree of hydrolysis, illustrating an exceptional level of precision in the data fitting.

The experimental analyses performed under the optimal conditions obtained by mathematical modeling demonstrate the correctness of the model. Under these working conditions, a chromium extraction yield of 97.6% and a degree of collagen hydrolysis of 0.4% were obtained. This proves that the mathematical model describes the process very well.

## Figures and Tables

**Table 1:** Range and levels of experimental parameters.

Variable	Name	Levels		
		−1	0	1
A	Oxalic acid concentration (%)	2	5	8
B	Contact time (min)	50	150	250
C	Temperature (°C)	30	60	90

**Table 2:** ANOVA for postulated model.

Source	Sum of Squares	df	Mean Square	F-Value	*p*-Value	
Model	9730.85	9	1081.21	86.45	<0.0001	Significant
A—Oxalic acid concentration	5507.78	1	5507.78	440.40	<0.0001	Significant
B—Contact time	827.23	1	827.23	66.15	<0.0001	Significant
C—Temperature	1835.57	1	1835.57	146.77	<0.0001	Significant
AB	1.27	1	1.27	0.1012	0.7597	
AC	11.09	1	11.09	0.8867	0.3777	
BC	1.88	1	1.88	0.1501	0.7100	
A^2^	81.75	1	81.75	6.54	0.0377	Significant
B^2^	715.00	1	715.00	57.17	0.0001	Significant
C^2^	614.81	1	614.81	49.16	0.0002	Significant
Residual	87.54	7	12.51			
Lack of Fit	87.54	3	29.18			
Pure Error	0.0000	4	0.0000			
Cor Total	9818.39	16				
Std. Dev.	3.54			R^2^		0.9911
Mean	52.66			Adj. R^2^		0.9796
CV (%)	6.72			Pred. R^2^		0.8573
				Adeq precision	30.5174	

**Table 3:** Criteria constraints and optimal solutions.

Name	Goal	Lower Limit	Upper Limit	Solution	Desirability
A: Oxalic acid concentration	is in range	2	8	7.36061	1
B. Contact time	is in range	50	250	122.48	1
C: Temperature	is in range	30	90	49.2335	1
Extraction yield (%)	maximize	10.12	89.23	73.1673	1

**Table 4:** ANOVA for postulated model.

Source	Sum of Squares	df	Mean Square	F-Value	*p*-Value	
Model	14,596.98	9	1621.89	37.01	<0.0001	significant
A—Oxalic acid concentration	203.01	1	203.01	4.63	0.0684	
B—Contact time	90.86	1	90.86	2.07	0.1931	
C—Temperature	9167.93	1	9167.93	209.22	<0.0001	significant
AB	0.0090	1	0.0090	0.0002	0.9890	
AC	170.43	1	170.43	3.89	0.0892	
BC	121.99	1	121.99	2.78	0.1391	
A^2^	32.10	1	32.10	0.7326	0.4204	
B^2^	107.11	1	107.11	2.44	0.1619	
C^2^	4646.25	1	4646.25	106.03	<0.0001	significant
Residual	306.74	7	43.82			
Lack of Fit	306.74	3	102.25			
Pure Error	0.0000	4	0.0000			
Cor Total	14,903.72	16				
Std. Dev.	6.62			R^2^		0.9794
Mean	17.83			Adj. R^2^		0.9530
CV (%)	37.13			Pred. R^2^		0.6707
				Adeq precision		17.1528

**Table 5:** Criteria constraints and optimal solutions.

Name	Goal	Lower Limit	Upper Limit	Solution	Desirability
A: Oxalic acid concentration	is target	2	8	7.36061%	1
B: Contact time	is target	50	250	122.48 min	1
C: Temperature	is in range	30	90	57.9198 °C	1
Hydrolysis degree (%)	is in range	0.38	89.23	0.38004%	1

## Data Availability

The original contributions presented in this study are included in the article/Supplementary Material. Further inquiries can be directed to the corresponding authors.

## Supplementary Materials

The following supporting information can be downloaded at: https://www.mdpi.com/article/10.3390/polym17172319/s1, Figure S1: Desirability plots of hydrolysis degree (%).
