# Supplementary material for: Optimization of Chromium Removal Conditions from Tanned Leather Waste for Collagen Valorization
Source: Polymers (Basel). 2025 Aug 27;17(17):2319. doi: 10.3390/polym17172319 (PMC12431349; doi:10.3390/polym17172319)
Supplement: Supplementary file 1 [file polymers-17-02319-s001.zip › polymers-3796426-supplementary.pdf]

Supplementary data

Optimization of chromium removal conditions from tanned leather waste

Ana-Maria Nicoleta Codreanu (Manea) [1], Daniela Simina Stefan [2]*, Lidia Kim [3], Ionut Cristea [3], Rachid Aziam [4]*

[1] Faculty of Chemical Engineering and Biotechnology, National University of Science and Technology Politehnica Bucharest, Polizu Street, No 1-7, ZIP Code 011061, Bucharest, Romania, e-mail: anamariacodreanu99@yahoo.com< A.M.C..

[2] Faculty of Chemical Engineering and Biotechnology, National University of Science and Technology Politehnica Bucharest, Department of Analytical Chemistry and Environmental Engineering, Polizu Street, No 1-7, ZIP Code 011061 Bucharest, Romania; daniela.stefan@upb.ro, D.S.S.

[3] National Research and Development Institute for Industrial Ecology, Drumul Podu Dambovitei Street, No 57-73, ZIP Code 060652, Bucharest, Romania; lidia.kim@incdecoind.ro, L.K., and ionut.cristea@incdecoind.ro, I.C.

[4] Laboratory of Applied Chemistry and Environment, Department of Chemistry, Faculty of Sciences, Ibnou Zohr University, BP 8106 Agadir, Morocco; rachid.aziam@edu.uiz.ac.ma, R.A.

* Correspondence: daniela.stefan@upb.ro, D.S.S., and rachid.aziam@edu.uiz.ac.ma, R.A.

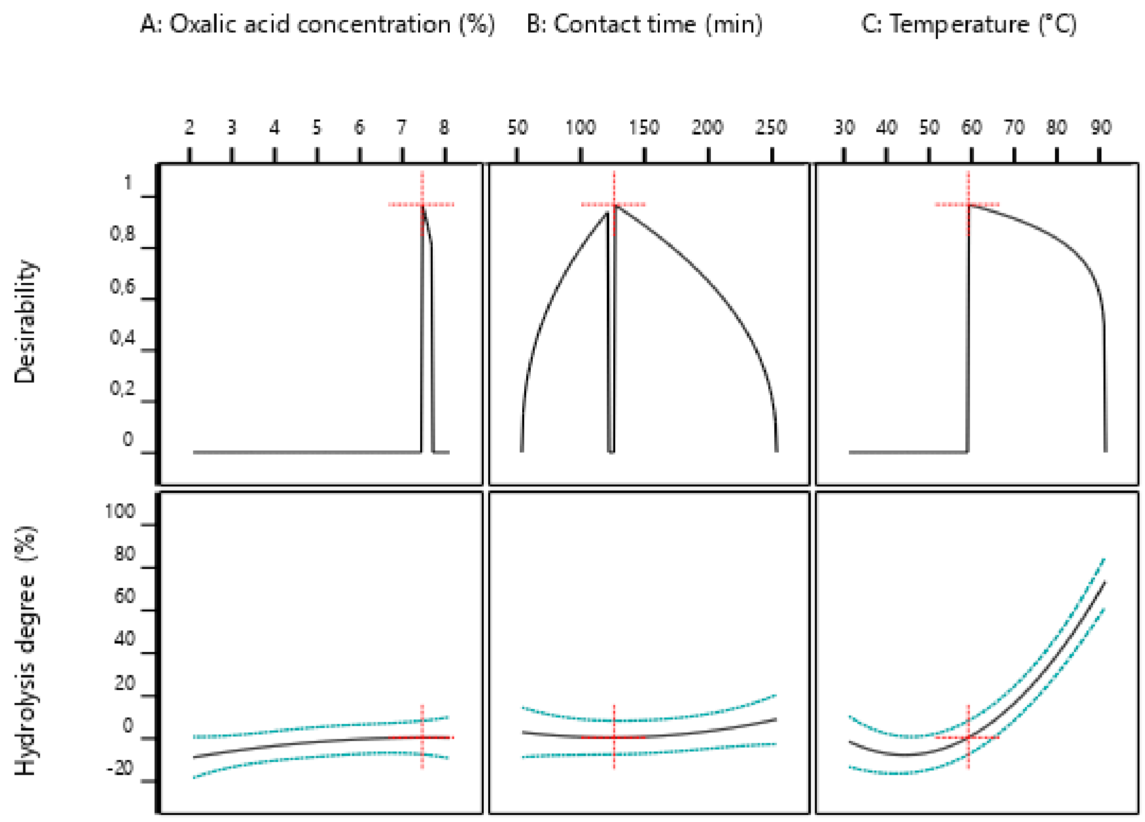

Figure S1. Desirability plots of Hydrolysis degree (%).
